# Reliability of synovial fluid alpha-defensin and leukocyte esterase in diagnosing periprosthetic joint infection (PJI): a systematic review and meta-analysis

**DOI:** 10.1186/s13018-019-1395-3

**Published:** 2019-12-19

**Authors:** Yisheng Chen, Xueran Kang, Jie Tao, Yunpeng Zhang, Chenting Ying, Weiwei Lin

**Affiliations:** 1 Department of Orthopedics, Shanghai General Hospital, Shanghai Jiao Tong University School of Medicine, Shanghai, China; 2 Department of Otolaryngology-Head and Neck Surgery, Shanghai Ninth People’ s Hospital, Ear Institute, Shanghai Key Laboratory of Translational Medicine on Ear and Nose Diseases, Shanghai Jiao Tong University School of Medicine, Shanghai, China; 3 Department of Orthopedics, Shanghai General Hospital, Shanghai Jiao Tong University School of Medicine, Shanghai, China; 4 Department of Neurosurgery, The First Affiliated Hospital of Wenzhou Medical University, Wenzhou, Zhejiang China

**Keywords:** α-Defensin, Leukocyte esterase, Periprosthetic joint infection, Meta

## Abstract

**Background:**

Synovial fluid proteins had been applied as diagnostic biomarkers for periprosthetic joint infection (PJI) in recent research papers. Thus, this meta-analysis aimed to estimate the diagnostic efficiency of synovial fluid α-defensin and leukocyte esterase (LE) for PJI.

**Methods:**

We conducted our systematic review by searching the keywords in online databases such as PubMed, Embase, Cochrane, Elsevier, Springer, and Web of Science from the time of database inception to October 2018. Inclusion criteria were as follows: patients who have undergone knee, hip, or shoulder joint replacements; α-defensin or leukocyte esterase (LE strip) of synovial fluid was detected as the biomarker for PJI diagnosis; and Musculoskeletal Infection Society (MSIS) or utilizing a combination of clinical data was considered as the gold standard. Diagnostic parameters including sensitivity, specificity, diagnostic odds ratio (DOR), and area under the summary of receiver operating characteristics curve (AUSROC) were calculated for the included studies to evaluate the synovial fluid α-defensin and LE for PJI diagnosis.

**Results:**

After full-text review, 28 studies were qualified for this systematic review, 16 studies used α-defensin and the other 12 were conducted using LE strip. The pooled sensitivity, specificity, and DOR of LE strip were 87% (95% CI 84–90%), 96% (95% CI 95–97%), and 170.09 (95% CI 97.63–296.32), respectively, while the pooled sensitivity, specificity, and DOR of α-defensin were 87% (95% CI 83–90%), 97% (95% CI 96–98%), and 158.18 (95% CI 74.26–336.91), respectively. The AUSROC for LE strip and α-defensin were 0.9818 and 0.9685, respectively.

**Conclusion:**

Both LE strip and α-defensin of synovial fluid provide rapid and convenient diagnosis for PJI. Sensitivity of α-defensin and LE strip are the same, while both these two methods have high specificity in clinical practice.

## Introduction

Periprosthetic joint infection (PJI) is a challenging problem that exerts significantly negative influence on patients’ life after total joint arthroplasty [1, 2]. For a painful joint after surgery, the ability to differentiate between PJI and aseptic loosening is of great significance since the treatment for these two situations are completely different. The latter situation requires a second-stage surgery to eradicate the infecting organisms [3].

Traditional routine test includes white blood cell (WBC) number, erythrocyte sedimentation rate (ESR), serum C-reactive protein (CRP), and synovial fluid culture, all of which are non-specific for PJI [4]. In addition, several orthopedic associations have established clinical guidelines based on consensus approaches, expert opinions, and reviews [5]. The American Academy of Orthopedic Surgeons (AAOS) guideline, which was released in 2010 [6], and Musculoskeletal Infection Society (MSIS) guideline [7, 8] helped to standardize and facilitate the diagnostic process for PJI. In 2018, a new definition of PJI was released [8] including several synovial fluid biomarkers, for instance, leukocyte esterase (LE) and α-defensin. Although both these two guidelines provide clinicians a standard for PJI diagnosis, they incorporate several criteria, which make them still difficult to execute in daily clinical practice. Thus, if a single or a combination of two or three tests could accurately diagnose PJI, the diagnostic efficient would be greatly improved in clinical practice and help clinicians to make next stage treatment plan. α-Defensin and LE strip are two biomarkers that are studied most widely for PJI diagnosis.

α-Defensin is an antimicrobial peptide which originates from neutrophils after its response to pathogens [9]. It has been reported that an α-defensin test can identify culture-negative infections [10]. Through interacting with the pathogens’ cell membrane, it can lead to depolarization and rapid kill of the pathogen [11, 12]. In addition, α-defensin are not influenced by antibiotic administration for the treatment of PJIs before diagnostic evaluation. It has been reported that α-defensin level did not suffer from a decrease with antimicrobial administration [13]. Compared with the MSIS criteria, which requires a serious of laboratory parameters, α-defensin immunoassay could make the diagnosis of PJI more simple and effective. Drago et al. [14] has reported that the specificity of α-defensin immunoassay is quite high for excluding the PJI after total hip arthroplasty (THA) and/or total knee arthroplasty (TKA). In addition, increased α-defensin level may be due to reasons other than a periprosthetic infection. The advantages of this test include its convenience and standardization, while a disadvantage is its relatively high cost per test [15].

Leukocyte esterase (LE) is an enzyme produced by activated neutrophils at the site of infection [16]. Detection of LE has traditionally been used to help diagnose urinary tract infection [17]. The LE in synovial fluid is detected by colorimetric strip tests through reactions, which produces a color change. Advantages of this test include quickness, convenience, and low cost. Due to its convenience, LE strip has been studied during the past several years for diagnosis of PJI. However, several issues has been aroused since then, such as the color change of the strip is frequently affected by blood and the cutoff value for PJI is not in consensus. Thus, more research is still required to solve these problems.

Due to the great heterogeneity of diagnostic criteria, protocols, and sample sizes in publicized papers, the diagnostic utility of these synovial fluid biomarkers has no clear consensus. With the increased use of synovial fluid biomarkers, the diagnostic efficiencies, economic advantages, and limitations have to be taken into consideration. Thus, we conducted a systematic review to summarize studies related to α-defensin and LE. We conducted a meta-analysis to investigate the diagnostic accuracy of these two methods in PJI. We aim to compare the diagnostic efficiency of these two most frequently used methods in clinical practice and provide clinicians with more accurate evidence.

## Material and methods

Data from the selected studies were extracted, and eligible studies were assessed by means of the revised Quality Assessment of Diagnostic Accuracy Studies (QUADAS-2) criteria [18]. Statistical analysis, evidence synthesis, and report compilation were carried out as the steps below. We strictly adhered to standards of the Preferred Reporting Items for Systematic Reviews and Meta-Analyses (PRISMA) in reporting the findings of this review (Additional file 1: Table S1 for PRISMA detailed checklist).

### Search strategy

We searched the electronic databases including PubMed, Embase, Web of Science, the Cochrane Library, and Science Direct since the release of MSIS definition to September 2018. Vocabulary and syntax were adjusted according to different databases. We used key words or Mesh words as follows: “periprosthetic joint infection” or “prosthesis-related infections” to represent the disease, “synovial fluid” or “fluid, synovial” to represent the source of our target biomarker, and “α-defensin” or “alpha defensin” or “defensin” or “leukocyte esterase” as our target index.

### Study selection

Screening was performed as follows. Two researchers firstly independently reviewed the title and abstract of each assay to select papers, which require full-text screening. In the initial stage of the screening, ten articles should be used to confirm the agreement between the researchers. When confronted with disagreements, two researchers had to come to a consensus about the screening standard. After full-text screening, a list of reasons for exclusion was performed.

Inclusion criteria were as follows: patients who have undergone knee, hip, or shoulder joint replacements; 1 ml synovial fluid had to be aspirated for study; synovial fluid α-defensin or LE strip was determined as the biomarker for PJI diagnosis; Musculoskeletal Infection Society (MSIS) or utilizing a combination of clinical data was considered as the gold standard; and sufficient data could be extracted for the construction of a 2 × 2 contingency table. Studies that lacked sensitivity and specificity values were also excluded.

### Quality assessment

The methodological quality of the included studies was appraised by an adapted version of the QUADAS-2, which are composed of four key domains (patient selection, index test, reference standard, and flow and timing). Signaling questions were applied to evaluate the risk of bias and clinical applicability. These questions were responded as “yes” for low risk of bias/concerns, “no” for high risk of bias/concerns, or “unclear”.

### Data extraction

The detailed information of qualified studies was extracted: (i) study characteristics including author, year of publication, country, design, and sample size; (ii) population characteristics including patients’ mean age, sex, location of joint replacement, and body mass index (BMI); (iii) intervention characteristics including method of sampling, method of measuring, and threshold; (iv) gold standard including the text results based on the definition of PJI by MSIS; (v) outcomes of tested biomarkers such as number of false positive, true positive, false negative, and true negative; and diagnostic parameters such as sensitivity, specificity, positive likelihood ratio (LR+), and negative likelihood(LR−).

### Statistical analysis and heterogeneity assessment

For all the studies from which we constructed the 2 × 2 table, pooled diagnostic parameters mentioned above were calculated through the bivariate model. The summarized receiver operating characteristic (SROC) curve was constructed.

In the diagnostic test, heterogeneity was commonly caused by the threshold effect, which was evaluated by Spearman’s correlation coefficient. If there were more than one threshold in an article, the threshold with the largest Yourdon index was chosen. The percentage of the total variation across studies was described by the *I*^2^ statistic, which indicated the existence of significant heterogeneity when the value exceeded 50%. The value of *I*^2^ ranges from 0 to 100%, with 0% implying no observed heterogeneity and larger values indicating increasing heterogeneity. For all effect estimates, a value of *p* < 0.05 was considered to be statistically significant. All analysis was conducted using Meta-disc software (version 14.0, Zamora et al., Madrid, Spain).

## Results

**Fig. 1:**
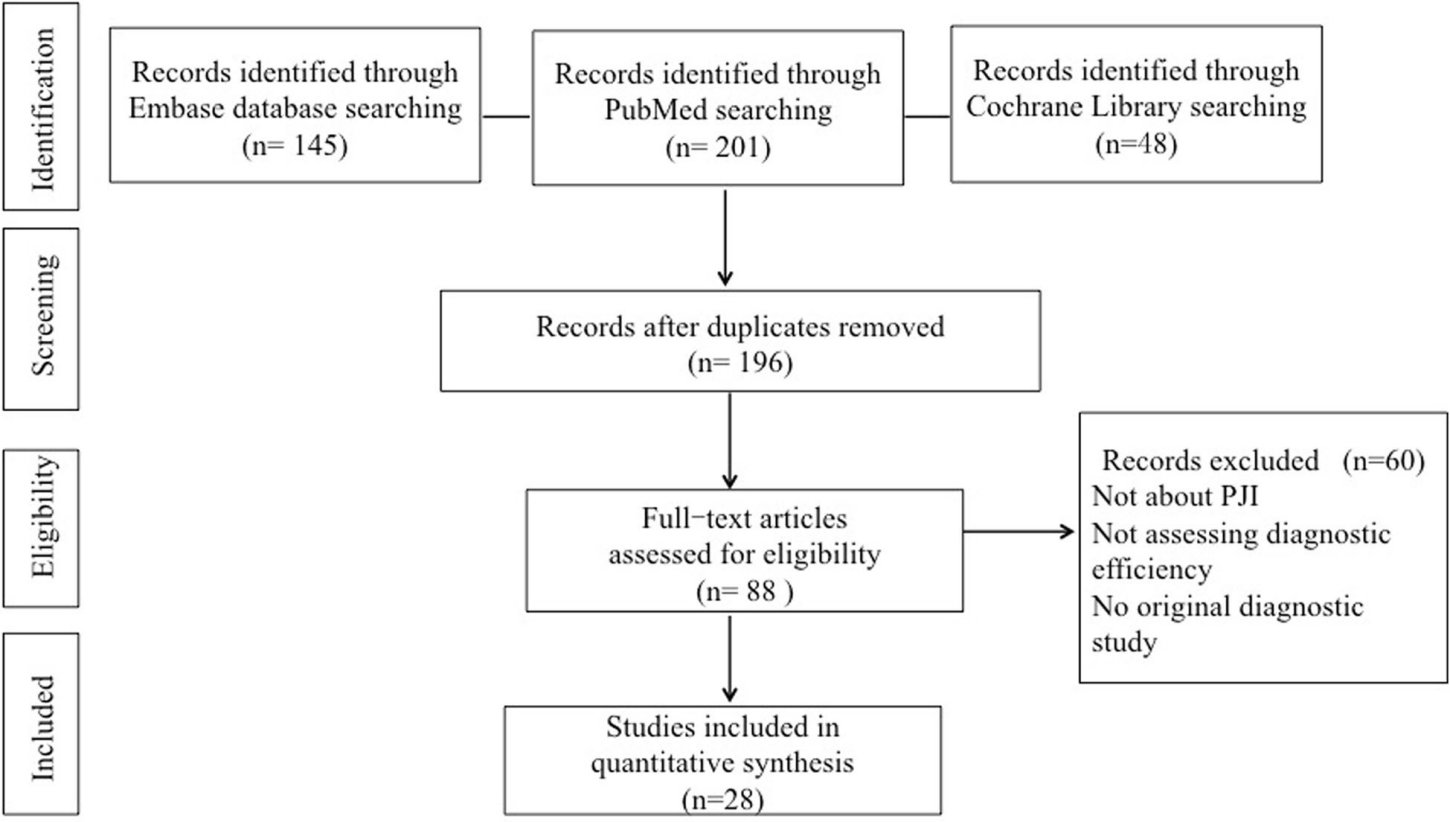
Flow chart of selection process for eligible studies

**Fig. 2:**
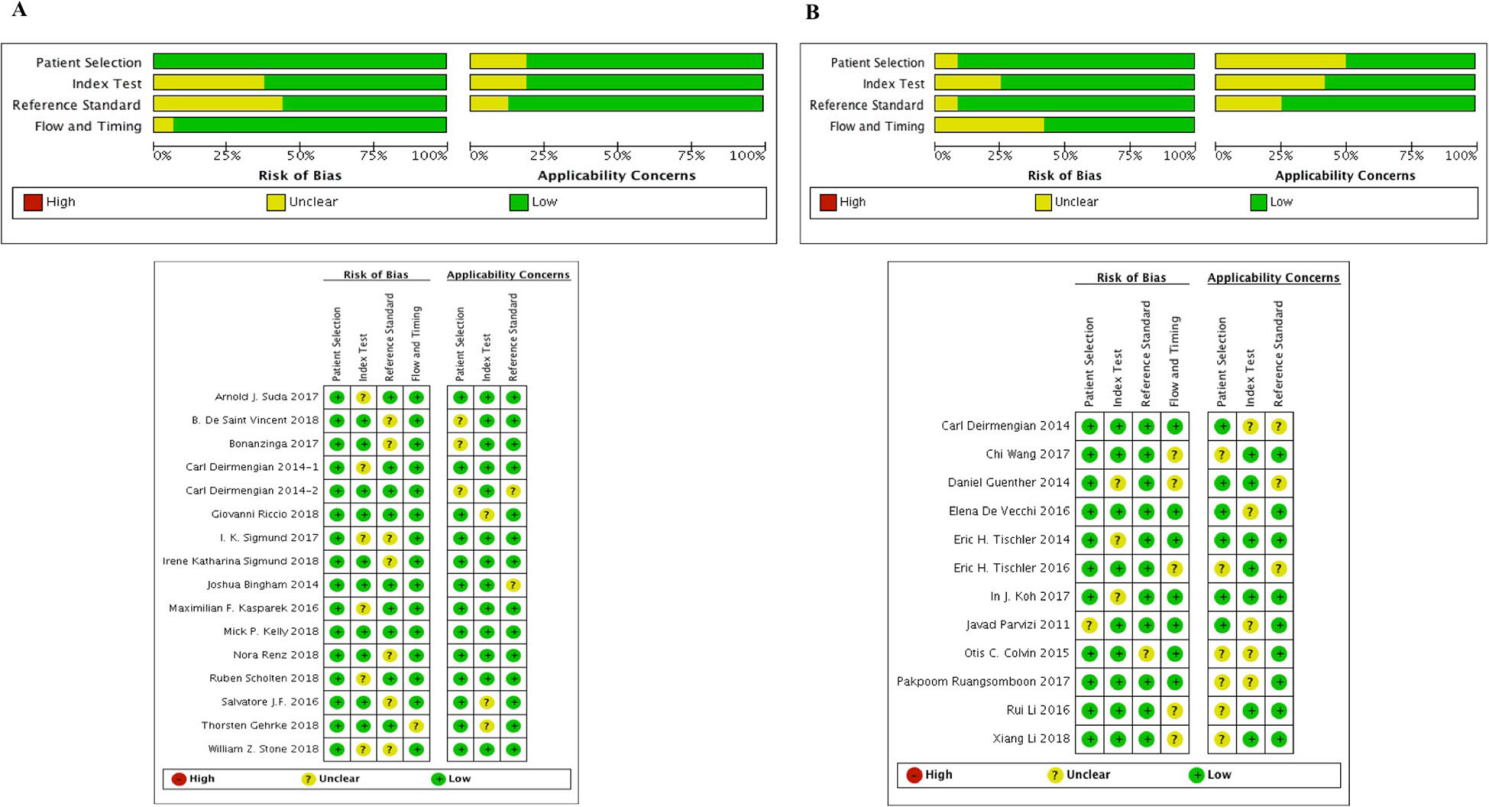
Quality assessment of included studies using QUADAS-2 tool criteria (**a** α-defensin, **b** LE strip)

Of the identified 394 articles, 196 of them were left for further screening after excluded the duplicates. One hundred eight articles were excluded after reading the title and abstract, reasons including inappropriate article type (reviews, comments, or letters). Then, the remaining 88 articles were read through, and 59 were unqualified due to incomplete data for systematic review. Among these included 29 articles, 16 articles [15, 19–33] explored the diagnostic accuracy of α-defensin for PJI, while the remaining 12 studies [34–45] explored the diagnostic accuracy of LE strip for PJI. The flow diagram is illustrated in Fig. 1. QUADAS-2 quality assessment for the included studies is shown in Fig. 2.

A total of 1547 patients who applied α-defensin and 1384 patients who applied LE strip for diagnosis of PJI were included in this meta-analysis. Among the included studies, 20 were conducted prospectively and the other nine retrospectively. The optimal cutoff value for α-defensin was pre-specified in four studies, varying as 7.72 [15], 4.8 [36], and 5.2 [20, 22] mg/l, respectively, with enzyme-linked immunosorbent assay (ELISA), while the other 12 studies used lateral flow test strip to determine positive result without cutoff value. Detailed characteristics of individual study were summarized in Table 1 (α-defensin) and Table 2 (LE strip).

**Table 1:** Characteristics of 16 studies applying alpha-defensin for meta-analysis

Study, year	Country	Participants (M/F)	Median age (range, years)	Study design	Detection method	Assay platform	Cutoff value	Gold standard
Bingham et al. 2014	USA	UA	UA	R	ELISA	Synovasure (CD Diagnostics)	7.72 mg/L	MSIS
Deirmengian et al. 2014-1	USA	70/79	65 (41–89)	P	ELISA	Hycult Biotech (Uden, the Netherlands)	5.2 mg/L	MSIS
Deirmengian et al. 2014-2	USA	44/51	66 (41–86)	P	ELISA	Hycult Biotech (Uden, The Netherlands)	4.8 mg/L	MSIS
Frangiamore SJ 2016	USA	53/63	63 (51–79)	P	ELISA	Synovasure (CD Diagnostics)	5.2 mg/L	MSIS
Kasparek et al. 2016	USA	UA	71 (41–91)	R	Lateral flow test	Synovasure (CD Diagnostics)	UA	MSIS
Suda et al. 2017	Germany	17/11	67.7 (39–88)	P	Lateral flow test	Synovasure™ PJI Test (Zimmer, Warsaw, IN)	UA	MSIS
Bonanzinga et al. 2017	Germany	66/90	UA	P	Immunoassay	Synovasure (CD Diagnostics)	UA	MSIS
Sigmund et al. 2017	Austria	22/28	65 (20–89)	P	Lateral flow test	Synovasure	UA	MSIS
de Saint Vincent et al. 2018	France	24/15	UA (35–78)	P	Lateral flow test	Synovasure™, (Zimmer, Warsaw, IN)	UA	MSIS
Kelly et al. 2018	USA	21/18	64 (33–88)	R	Immunoassay	CD Diagnostics	UA	MSIS
Scholten et al. 2018	Netherlands	22/15	66 (51–81)	P	Lateral flow test	Synovasure™, (Zimmer, Warsaw, IN)	UA	Culture
Gehrke et al. 2018	Germany	77/114	UA	P	Lateral flow test	Synovasure kit	UA	MSIS
Sigmund et al. 2018	Germany	38/33	70 (41–85)	R	Lateral flow test/ELISA	Synovasure kit (Zimmer Biomet)/Synovasure™ (CD Diagnostics)	UA	MSIS/EBJIS/IDSA
Riccio et al. 2018	Italy	30/45	68.7 (57–79)	R	Lateral flow test	Synovasure (CD Diagnostics)	UA	MSIS
Stone et al. 2018	USA	78/105	65.7 (34–91)	P	Microarray	Synovasure (CD Diagnostics)	UA	MSIS
Renz et al. 2018	Germany	61/106	70 (41–94)	P	Lateral flow test	Synovasure kit (Zimmer Biomet)	UA	MSIS

*UA* unavailable, *P* prospective study, *R* for retrospective study, *ELISA* enzyme-linked immunosorbent assay, *EBJIS criteria* European Bone and Joint Infection Society criteria, *IDSA* Infectious Diseases Society of America

**Table 2:** Characteristics of 13 studies applying leukocyte esterase (LE) strip for meta-analysis

Study, year	Country	Participants (M/F)	Median age (range, years)	Standard reference	Study design	Assay platform	Cutoff value
Parvizi et al. 2011^&^	USA	UA (108)	UA	Own institute^#^	P	Chemstrip 7 urine test strip (Roche Diagnostics, Indianapolis, Indiana)	++ (+)*
Tischler et al. 2014	USA	90/99	63 (22–90)	MSIS	P	UA	++ (+)*
Deirmengian et al. 2014^ζ^	USA	28/18	63/67	MSIS	R	Chemstrip 7 urine test strip (Roche Diagnostics, Indianapolis, IN)	++/+
Guenther et al. 2014^&^	Germany	UA (353)	67 (56–78)	MSIS	P	Roche Diagnostics GmbH, Mannheim, Germany	++/+
Colvin et al. 2015^&^	USA	27/30	69.1 (31–91)	AAOS	P	Chemstrip 7 urine test strips (Roche Diagnostics, Indianapolis, IN)	++
Tischler et al. 2016	USA	30/31	64.1 (45–80)	MSIS	P	UA	++
De Vecchi et al. 2016	Italy	UA (129)	64 (17–88)	MSIS	P	Dirui Industrial Co Ltd., China	++/+
Ruangsomboon et al. 2017	Thailand	11/35	69 (61–77)	ICM criteria	R	Chemstrip 10 urine test strip; Roche Diagnostics, Indianapolis, Indiana	++
Koh et al. 2017	Korea	13/47	71 (50–85)	MSIS	P	AUTION ELEVEN, ARKRAY, Kyoto, Japan; Clinitek 500, Siemens, Munich, Germany; and Urisys 2400, Roche Diagnostics, Mannheim, Germany	++
Li et al. 2017	China	27/36	57.2 (22–80)	MSIS	P	Comber 10 Test M Roche (Germany)	++
Wang et al. 2017	China	UA	63 (51–75)	MSIS	R	Combur10 TestM Roche, Germany; AUTION Sticks, Arkray, Kyoto, Japan	++
Li et al. 2018	China	81/117	62 (48–76)	MSIS	P	AUTION Sticks, Arkray, Kyoto, Japan	++/+

*UA* unavailable, *P* prospective study, *R* retrospective study, *ELISA* enzyme-linked immunosorbent assay, *ICM* International Consensus Meeting

*Both ++ and ++/+ as cutoff value were analyzed for the sensitivity, specificity, positive predictive value, and negative predictive value

^#^Similar to MSIS

^&^Blood samples excluded

^ζ^Both bloody and non-bloody samples were analyzed for the sensitivity, specificity, positive predictive value, and negative predictive value, but only non-bloody samples were included in the meta-analysis

**Fig. 3:**
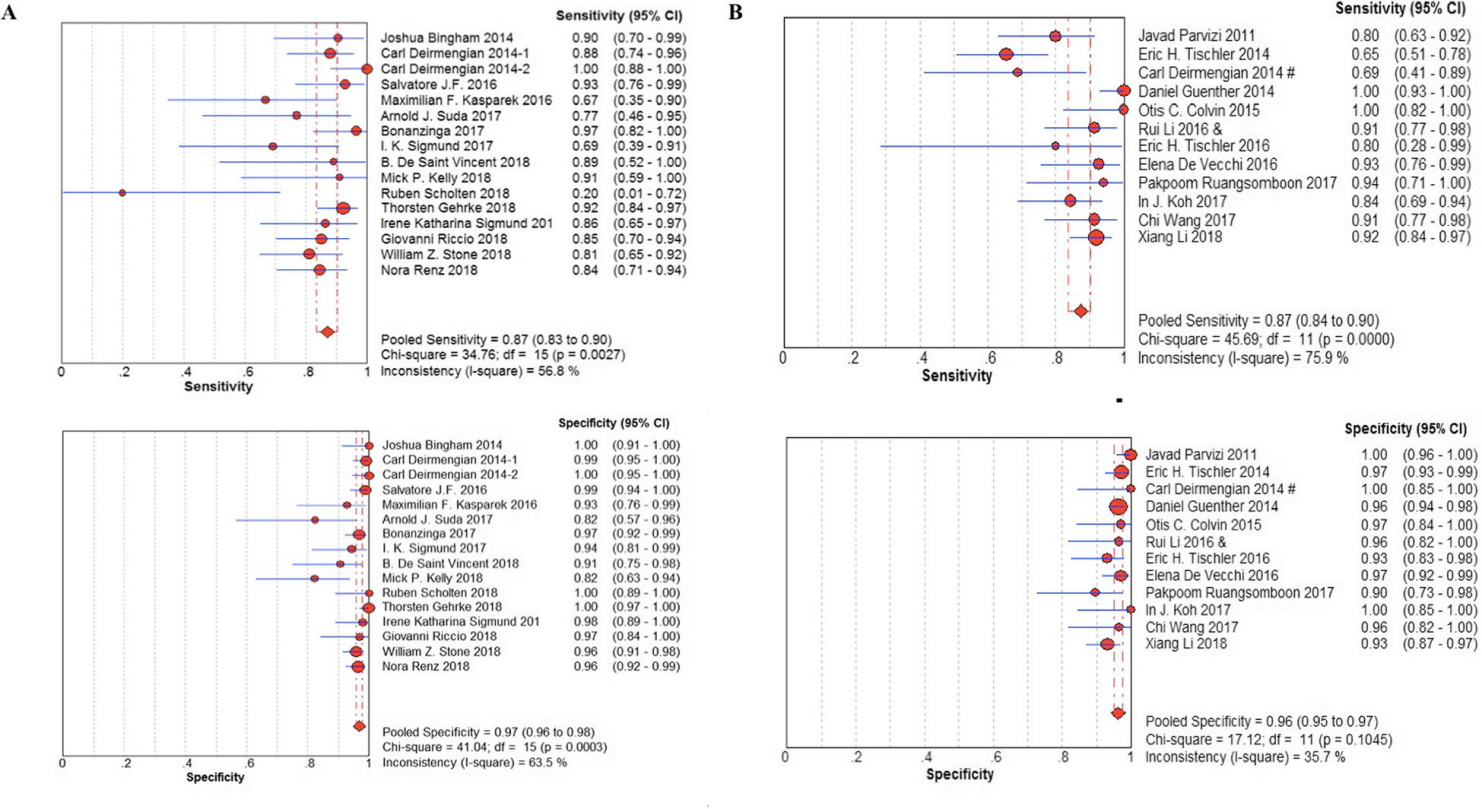
Pooled sensitivity and specificity for the included studies with the associated 95% confidence interval (**a** α-defensin, **b** LE strip)

**Fig. 4:**
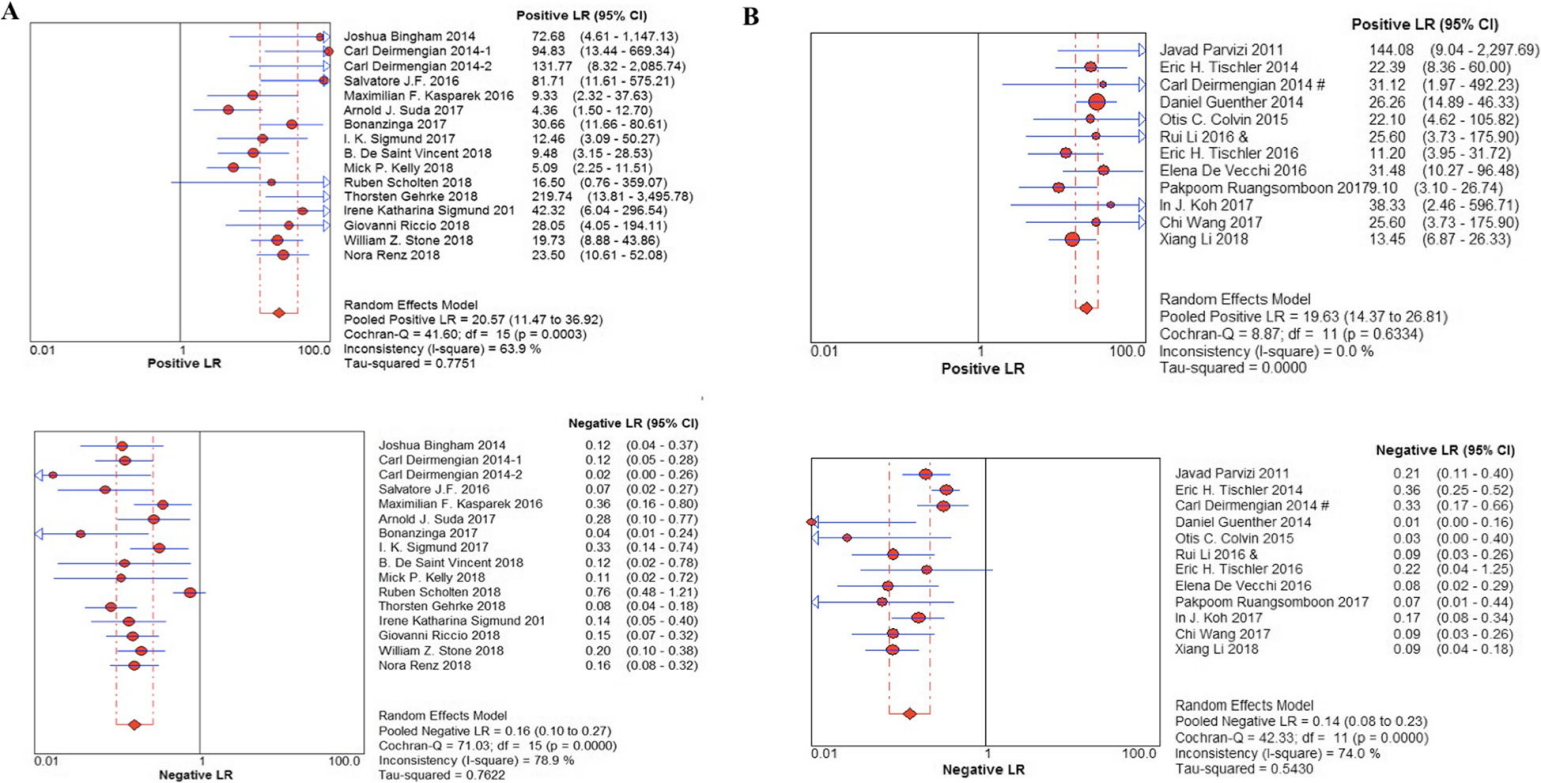
Positive likelihood ratio (PLR) and negative likelihood ratio (NLR) for the included studies with the associated 95% confidence interval (**a** α-defensin, **b** LE strip)

**Fig. 5:**
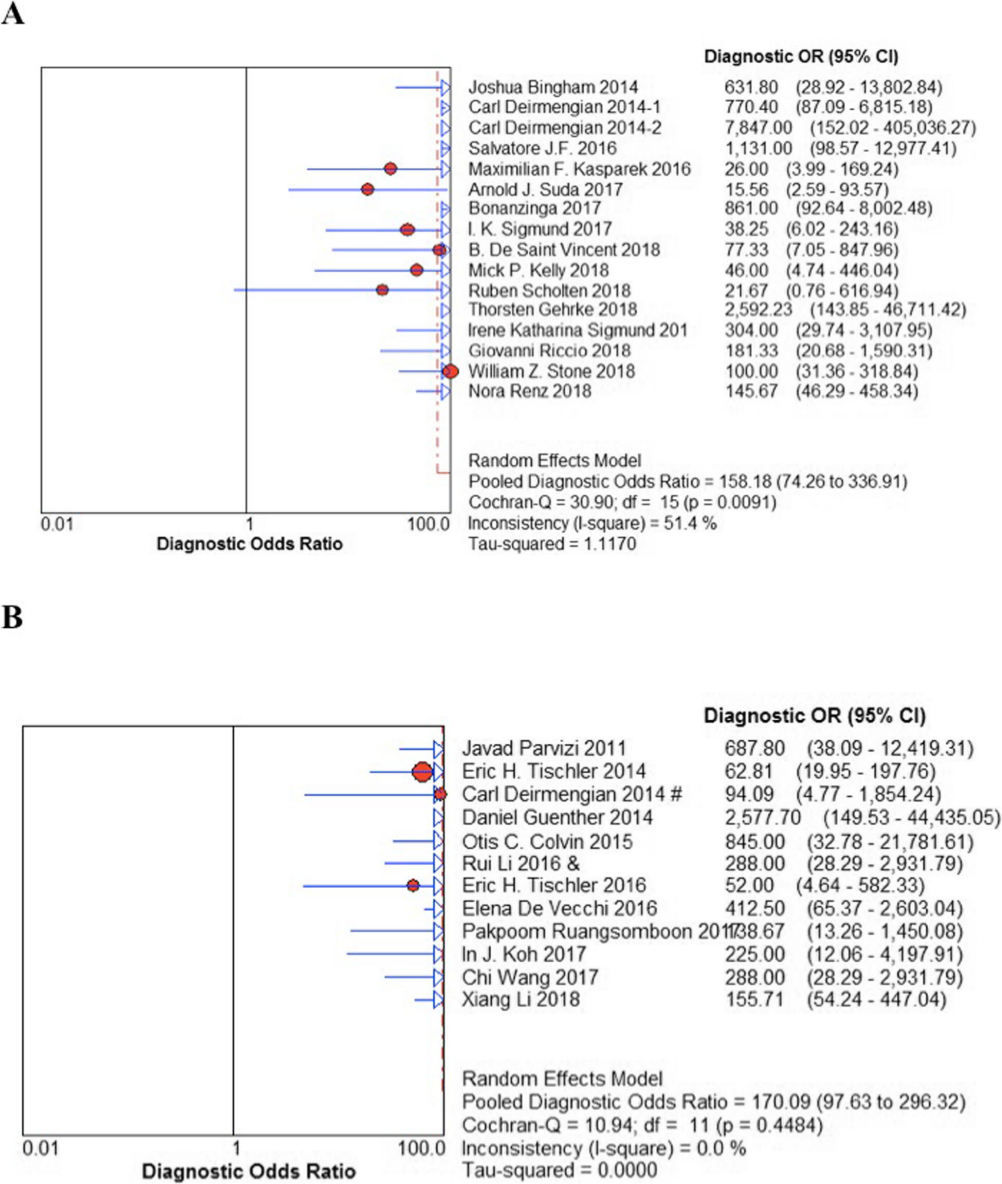
Diagnostic OR for the included studies with the associated 95% confidence interval (**a** α-defensin, **b** LE strip)

**Fig. 6:**
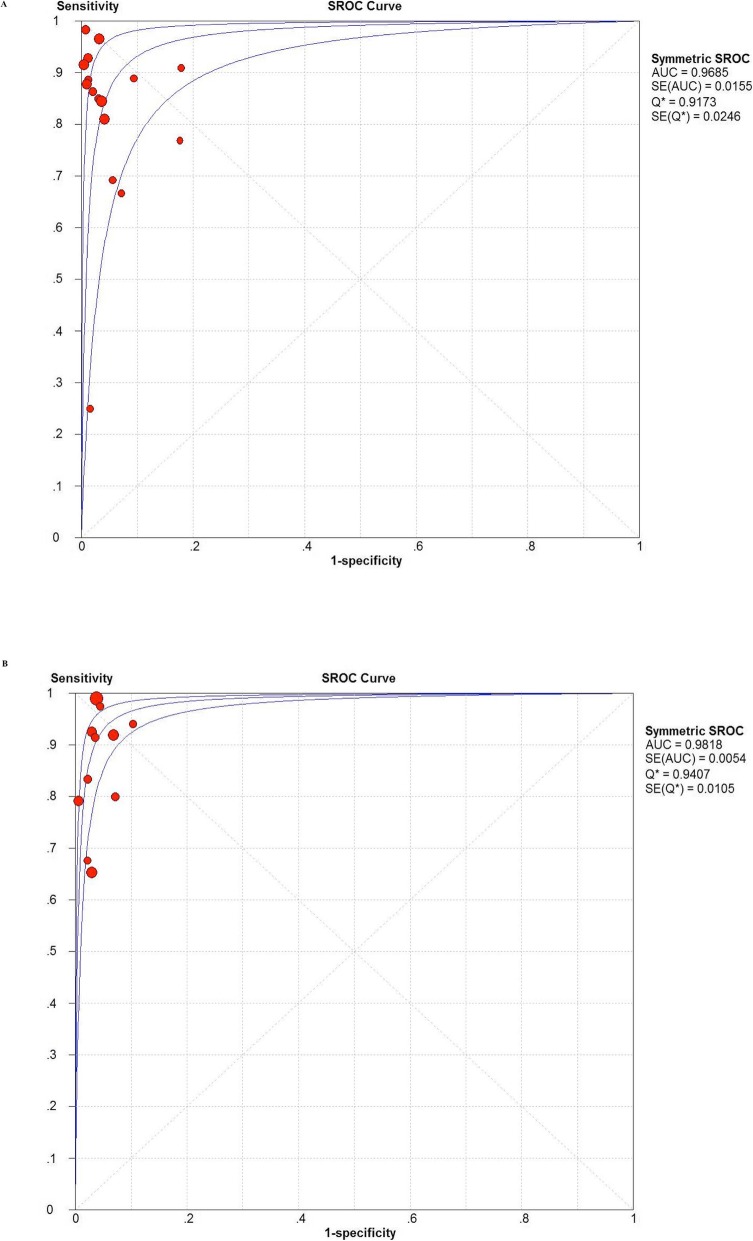
Summary receiver operating characteristic plot for the included studies with the associated 95% confidence region and the 95% prediction region (**a** α-defensin, **b** LE strip)

The pooled sensitivity and specificity of α-defensin were 87% (95% CI 83–90%) and 97% (95% CI 96–98%), respectively, while the pooled sensitivity and specificity of LE strip were 79% (95% CI 75–82%) and 96% (95% CI 95–97%), respectively (Fig. 3). The pooled positive likelihood ratio (PLR) and negative likelihood ratio (NLR) of α-defensin and LE strip are illustrated in Fig. 4. The pooled diagnostic odds ratio (DOR) of α-defensin and LE were 158.18 (95% CI 74.26–336.91) and 164.18 (95% CI 85.81–314.11), respectively (Fig. 5). The areas under the summary of receiver operating characteristics curve (SROC) for LE strip and α-defensin were 0.9826 and 0.9685, respectively (Fig. 6).

**Fig. 7:**
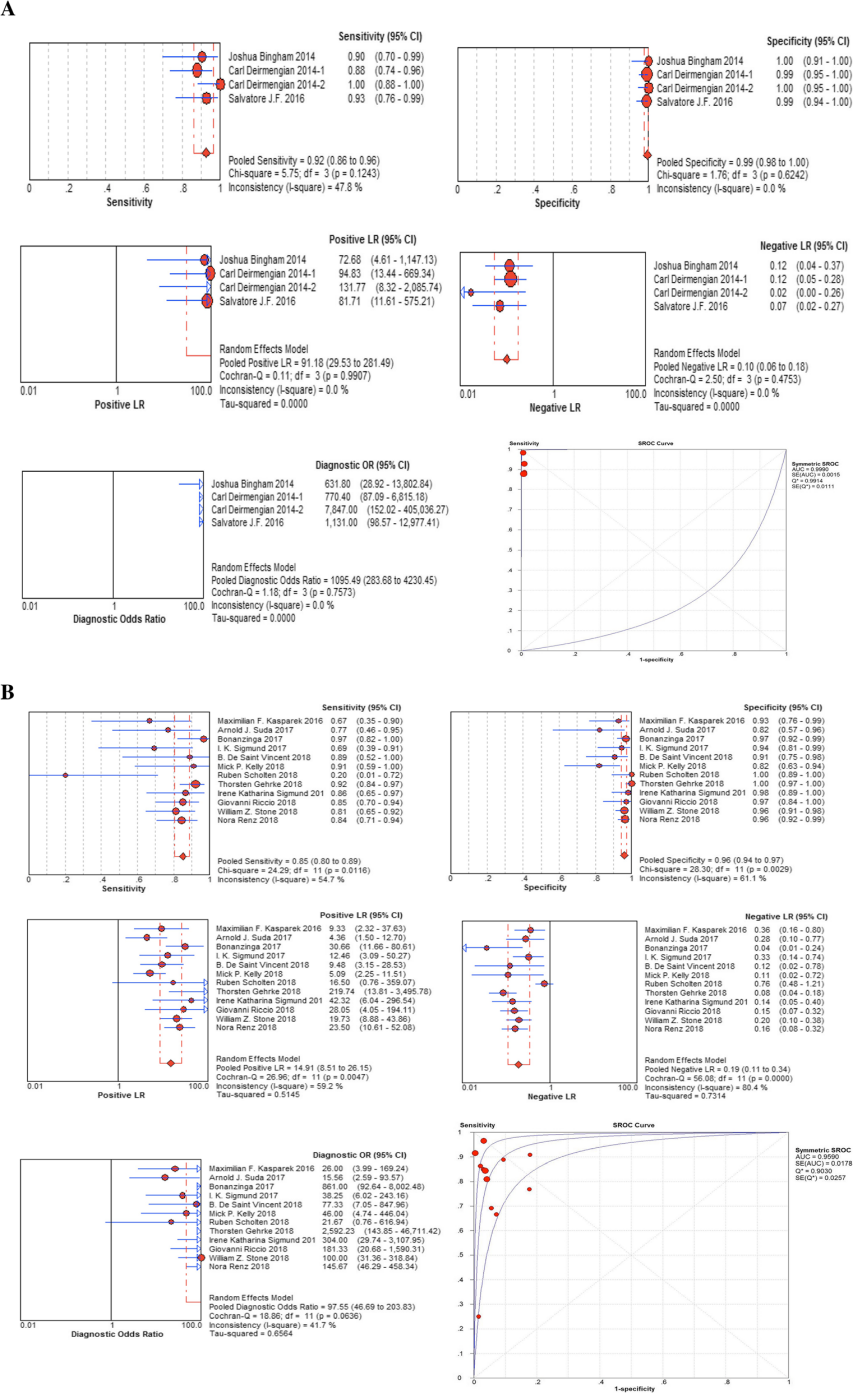
Pooled diagnostic parameters of enzyme-linked immunosorbert assay (ELISA) (**a**) and lateral flow test strip (**b**) for α-defensin A: enzyme-linked immunosorbert assay (ELISA), B: lateral flow test strip

There are two methods used for diagnosis of α-defensin (ELISA and lateral flow test strip), which have different sensitivities. Thus, we divided these included studies into two subgroups based on the methods used. The pooled diagnostic parameters are illustrated in Table 3. There was substantial heterogeneity among studies: the *I*^2^ statistics for sensitivity and specificity values of α-defensin were 56.8% and 63.5%, respectively, while the *I*^2^ statistics for sensitivity and specificity values of LE strip were 92% and 33.1%, respectively (Figure 7).

**Table 3:** Pooled diagnostic parameters of ELISA and lateral flow test strip for α-defensin

	ELISA	Lateral flow test strip
Number of studies	4	12
Sensitivity (95% CI)	92% (86–96%)	85% (80–89%)
Specificity (95% CI)	99% (98–100%)	96% (94–97%)
Positive likelihood ratio (95% CI)	91.18 (29.53–281.49)	14.91 (8.51–26.15)
Negative likelihood ratio (95% CI)	0.10 (0.06–0.18)	0.19 (0.11–0.34)
Diagnostic odds ratio (95% CI)	1095.49 (283.68–4230.45)	97.55 (46.69–203.83)
AUC	0.9990	0.9590

## Discussion

Our systematic review indicated that synovial fluid α-defensin can be used as a sensitive and specific biomarker in identifying PJI, while LE strip is slightly less sensitive but also extremely specific when compared with α-defensin. Both these two proteins perform better than other serological and synovial fluid markers (e.g., erythrocyte sedimentation rate [46], synovial fluid procalcitonin [47], synovial fluid interleukin-6 [48], synovial fluid CRP [49]) (Table 4). These biomarkers are frequently affected by other inflammatory diseases such as rheumatic arthritis and osteoarthritis. Therefore, novel biomarkers for PJI diagnosis arise. Our meta-analysis summarized the recently publicized articles related with the diagnosis of PJI with α-defensin or LE strip and included the above 29 studies, which in total contains over 3000 patients. Since all studies were publicized after 2011, which is the year that MSIS criteria had been bring into clinical practice, most of the included studies used MSIS as the gold standard. This greatly minimizes classification bias by using the “standard” based on the experience of clinicians in each hospital. Synovial fluid aspirated from patients who have undergone total joint replacement provides researchers with a perfect source of PJI diagnosis. In recent years, research on PJI diagnosis focused on synovial fluid, as it represents the local environment of infection, and diagnosis should be more sensitive than that of serum markers.

**Table 4:** Diagnostic values of other serum or synovial fluid biomarkers for PJI

Biomarker	Sensitivity (95% CI)	Specificity (95% CI)
Erythrocyte sedimentation rate (ESR)	86% (82.5–89%)	72.3% (70.4–74.2%)
Serum C-reactive protein (CRP)	86.9% (83.5–89.9%)	78.6% (86.9–80.3%)
Synovial fluid procalcitonin (PCT)	53% (24–80%)	92% (45–99%)
Synovial fluid CRP	92% (86–96%)	90% (87–93%)
Synovial fluid interleukin-6 (IL-6)	72% (63–80%)	91% (82–96%)

There are several host proteins in synovial fluid with antimicrobial activity, which play an important role in the response to pathogens elimination [50], among which α-defensin is considered of great clinical significance. Through searching the currently publicized essays, we found that α-defensin test was performed with Synovasure (CD Diagnostics), Synovasure™ PJI Test (Zimmer, Warsaw, IN), or Hycult Biotech (Uden, The Netherlands). Through “α-defensin flow later” test, synovial fluid is dropped onto the test device and then migrates to the buffering pad to further combine with the specific antibody. It takes about 10 min for the mixture to across the test line, and then the result is provided to the operator [51]. The main problem confronted with the orthopedic surgeons is that most of them lack the experience and training to evaluate the significance of quality control and proper documentation when using point of care test (POCT) assays.

Among the commonly used biomarkers in synovial fluid for PJI, the urinary LE test strip is inexpensive, convenient, and commercially available. However, since the LE test strip was originally developed for urinary testing, the characteristics in synovial fluid ought to be re-evaluated. According to our current meta-analysis, the main problem of LE strip is that the cutoff value is not determined since it is a colorimetric test and many factors including blood can greatly influence the result. Due to the high rate of blood interference, centrifugation should be performed on synovial fluid samples before they are dropped on the LE strip. The urinary LE tests strip sometime fails to detect LE enzymatic activity, even when there is abundant LE in the synovial fluid. It was attributed to the LE inhibitors in inflamed synovial fluid [52].

However, there are several limitations in our study. Firstly, although we have included 16 studies for α-defensin, the detection was based on mainly two companies: Synovasure™ PJI Test (Zimmer, Warsaw, IN) and Synovasure (CD Diagnostics). Since there is still no standard cutoff value for the diagnosis worldwide, different laboratories used different cutoff value to determine the PJI patients, which means large-scale prospective randomized trials are required to address this problem. Several studies of α-defensin came from the same research group [10, 20, 36], which might affect the generalization of our findings. In addition, none of the studies mentioned about blinding and the time point of sampling, which might potentially introduce selection bias. Last but not the least, according to the pooled data, the sensitivity, specificity, and AUC of ELISA were all higher than those of lateral flow test strip. However, the subgroup of ELISA contains merely four studies while the other group contains 12 studies. Thus, more studies with ELISA methods ought to be carried out to further confirm the diagnostic efficiency of this method.

In conclusion, based on this meta-analysis, α-defensin is substantially more expensive (US$760 per test) than LE strip (US$0.17 per test). However, α-defensin may be more sensitive in diagnosing PJI, and both tests may have played important roles in PJI diagnosis. Considering all these aspects, the α-defensin assay, although representing a convenient method for orthopedics, could not be used as the only marker to rule out PJI. Such situations happen in clinical practice, and it should be integrated with other MSIS criteria so that a more precise and accurate diagnosis could be obtained. Although there are still lots of work to do, our study demonstrated a solid foundation for clinicians to use these simple, prompt, and convenient detection methods to diagnose PJI accurately and efficiently. In the future, development of more simple and convenient point of care tests should be the focus of research efforts.

## Supplementary information

**Additional file 1.** PRISMA 2009 Checklist.

## Data Availability

All data generated or analyzed during this study are included in this published article.

## Abbreviations

AAOS: American Academy of Orthopedic Surgeons
AUSROC: Area under the summary of receiver operating characteristics curve
BMI: Body mass index
CRP: C-reactive protein
DOR: Diagnostic odds ratio
ELISA: Enzyme-linked immunosorbent assay
ESR: Erythrocyte sedimentation rate
LE: Leukocyte esterase
LR: Likelihood ratio
MSIS: Musculoskeletal Infection Society
PJI: Periprosthetic joint infection
QUADAS-2: Quality Assessment of Diagnostic Accuracy Studies
SROC: Summarized receiver operating characteristic
THA: Total hip arthroplasty
THA: Total knee arthroplasty
WBC: White blood cell
